# Radiolucent foreign body leading to complete small bowel obstruction: a diagnostic dilemma – a case report

**DOI:** 10.1097/MS9.0000000000001226

**Published:** 2023-10-02

**Authors:** Krishna K. Yadav, Ranjeet Ghimire, Sudan Subedi, Krishna Kandel, Rupesh K. Yadav, Bikal Ghimire, Jayant K. Shah

**Affiliations:** a Department of General Surgery, Tribhuvan University Teaching Hospital, Maharajgunj; b Maharajgunj Medical Campus, Institute of Medicine, Tribhuvan University; c Nepalese Army Institute of Health Science, Kathmandu, Nepal

**Keywords:** exploratory laparotomy, foreign body ingestion, radiolucent foreign body, small bowel obstruction

## Abstract

**Introduction and importance:**

Foreign body ingestion leading to luminal obstruction in both the small and large bowels is rare, especially in children. The authors present a case of a 7-year-old patient who presented with a small bowel obstruction caused by an ingested radiolucent foreign body. The previous herniotomy surgery 1 year back led to initial diagnostic confusion, highlighting the need for a broad differential diagnosis.

**Case presentation:**

A 7-year-old child with a history of herniotomy presented with symptoms of small bowel obstruction. Radiological imaging revealed a soft tissue mass mimicking a polyp or cystic lesion. During exploratory laparotomy, a cystic structure was discovered in the terminal ileum. The foreign body, identified as a fluid-filled balloon, was inaccessible to endoscopy and was gently maneuvered into the ascending colon. It was punctured and removed during on-table colonoscopy.

**Clinical discussion:**

This case underscores the challenges of diagnosing and managing luminal obstruction caused by radiolucent foreign bodies in children. The presence of previous surgery can mislead clinicians, necessitating a broad differential diagnosis. Radiological imaging played a crucial role in identifying the foreign body. Surgical intervention guided by an on-table colonoscopy allowed successful removal.

**Conclusion:**

Foreign body ingestion leading to luminal obstruction should be considered, even in cases with previous abdominal surgery. Radiological imaging aids in identification, and timely surgical intervention, guided by on-table colonoscopy, facilitates foreign body removal. Awareness of such cases is essential for optimal care in pediatric patients with luminal obstruction caused by foreign body ingestion.

## Introduction

Highlights

- A 7-year-old patient with a history of herniotomy presented with small bowel obstruction caused by an ingested radiolucent foreign body.
- Initial diagnostic confusion occurred due to the previous surgery, leading to a mistaken assumption of adhesions as the cause of the obstruction.
- Exploratory laparotomy revealed a cystic structure in the terminal ileum, which was identified as a fluid-filled balloon and successfully extracted.
- The case emphasizes the need to consider foreign body ingestion as a potential cause of luminal obstruction, even in patients with a history of previous surgeries.
- Radiological imaging and on-table colonoscopy played crucial roles in the diagnosis and management of the patient, highlighting the importance of a multidisciplinary approach.

Small bowel obstruction is a common surgical emergency due to mechanical or functional obstruction of the bowel^1^. Common presenting symptoms of small bowel obstruction are inability to pass stool and flatus, pain in the abdomen, nausea, vomiting, abdominal distension, and anorexia. The symptomatology of small bowel obstruction mimics a number of medical and surgical conditions^2^. Bowel obstruction can be classified as complete, partial, or closed loop obstruction. There is passage of flatus but not stool in partial bowel obstruction. A closed loop obstruction occurs when there is obstruction both proximally and distally in a segment of the bowel. Acute small bowel obstruction leads to serious complications ranging from volume depletion and electrolyte disturbances to bowel gangrene and perforation, thereby warranting surgical interventions^3^.

In 90% of cases, small bowel obstruction is caused by adhesions, hernias, and neoplasms. Foreign body ingestion is an uncommon cause of bowel obstruction, even in the pediatric population. Eighty to ninety percent of foreign bodies are spontaneously passed. Only 10% of foreign bodies may not pass the pylorus^4^. Frequent sites of obstruction due to a foreign body are the pylorus, c-loop of duodenum, and the ileocecal junction. Ten to twenty percent of ingested foreign bodies require endoscopic removal, and less than 1% need surgical extraction. There have been reports of bowel obstruction due to fruit seeds, phytobezoars^5^, gossypibioma^6^ and intragastric balloons^7^.

Accurate diagnosis and management of small bowel obstruction are critical for minimizing the morbidity and mortality associated with this condition. Prompt recognition of the cause of small bowel obstruction, including rare but potentially dangerous causes such as foreign body ingestion, can help guide appropriate intervention and improve patient outcomes.

This case report has been reported in line with the SCARE criteria^8^.

## Case presentation

A 7-year-old female presented to our hospital with complaints of pain in the abdomen and vomiting for 5 days. She had not passed stool and flatus for 3 days. Abdominal pain was colicky, generalized, and of moderate-to-severe intensity. Vomiting had occurred for 12 episodes in the last 3 days. It was non-projectile, bilious, and non-blood stained. Vomiting was aggravated by food intake, leading to the child’s refusal to eat. Her mother had noticed distension in her abdomen. She had not passed stool and flatus for 3 days. She had no history of fever, jaundice, or passage of red-colored stool. Before these symptoms, the child was otherwise healthy and had development comparable to her peers. She had undergone a herniotomy for left inguinal hernia 1 year back.

**Figure 1:**
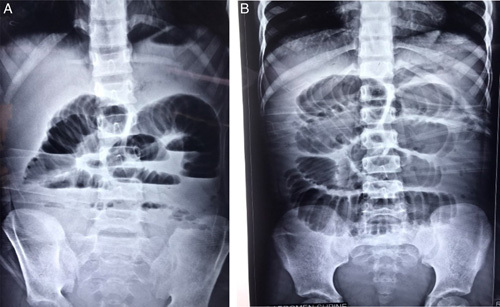
(A) Erect X-ray: abdomen showing air fluid level suggesting small bowel obstruction. (B) Supine X-ray: abdomen showing distended small bowel loops involving whole small bowel.

On physical examination, the patient appeared dehydrated, ill looking, and in pain. Her vitals were within normal limits. Generalized distension of the abdomen was present. The abdomen was soft to palpation; however, there was generalized tenderness without rebound tenderness. The X-ray of the abdomen showed multiple air fluid levels along with distended small bowel loops, suggesting small bowel obstruction, as shown in Figure 1A, B. Blood parameters were unremarkable. A provisional diagnosis of complete adhesive small bowel obstruction was made.

**Figure 2:**
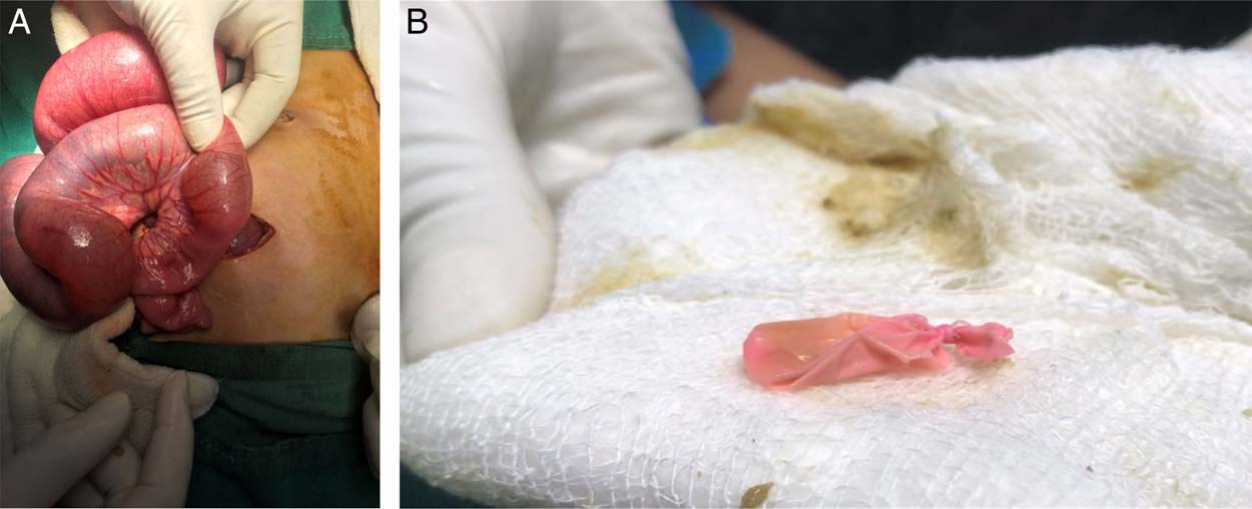
(A) Intraluminal foreign body of diameter ~3 cm. (B) Surgically removed fluid-filled balloon following deflation.

The patient was managed conservatively with nasogastric tube insertion, Foley’s catheterization, and intravenous dextrose normal saline. Injection ceftriaxone 500 mg was also given as a prophylactic antibiotic. After initial resuscitation, an emergency exploratory laparotomy was planned. Under general anesthesia, the abdomen was opened. Dilated ileal and jejunal loops with a transition point 40 cm proximal to the ileocecal valve were noted. No gangrenous segments were observed. A cystic swelling was noted over the transition point, which was mobile. The swelling was not attached to any underlying structure and was found to be a foreign body. The foreign body was milked through the ileocecal valve into the ascending colon as shown in Figure 2A. Dilated ileal and jejunal loops were decompressed through the duodeno-jejunal flexure. A colonoscopy was performed on the operating table, and the foreign body was identified to be a fluid-filled rubber balloon of 3 cm diameter approximately. It was ruptured and retrieved using endoscopic forceps, as shown in Figure 2B.

Postoperatively, the patient’s party was told about the operative findings. The patient admitted to having ingested a fluid-filled balloon 7 days back.

## Discussion

Adhesions are regarded as the most common cause of bowel obstruction in post-operative patients^7^. Ingestion of foreign body is not uncommon in the pediatric population. However, the diagnosis of small bowel obstruction may not be easily attributed to a foreign body, as the event is often unwitnessed and is usually denied by the patient^1^. The symptoms may also mimic a number of other conditions. The diagnosis may be further challenging when the ingested body is radiolucent. These situations require surgeons to conduct endoscopies of exploratory laparotomies to identify and extract the foreign body.

Sharp foreign bodies may injure the bowel wall and cause perforation. So immediate removal, either by endoscopy or surgery, is recommended. Smooth, soft, and round bodies almost always pass and do not cause obstruction. Obstruction caused upon swallowing such bodies may suggest the presence of some congenital anomalies such as duodenal stenosis, prolapsing duodenal diaphragm, and annular pancreas^9^.

This highlights the importance of proper supervision of children by parents. Children with developmental disorders or psychiatric illnesses require extra attention in this regard. On the clinician’s part, it is important to consider foreign body ingestion as a cause of luminal bowel obstruction, especially in the pediatric population given the popularity of plastic toys. Radiological investigations may not necessarily be beneficial for the diagnosis of radiolucent foreign bodies. However, systematic analysis of object shapes and densities by computed tomography (CT) may help in the identification of an ingested foreign body^10^. Proper history-taking and meticulous CT imaging and analysis may reduce the need for unnecessary surgical interventions and help guide endoscopic extractions.

## Conclusion

This case report on radiolucent foreign bodies causing small bowel obstruction highlights the importance of considering foreign body ingestion in pediatric patients. The diagnostic challenge, due to the unwitnessed nature of events and denial by patients, emphasizes the need for thorough history-taking and meticulous imaging analysis. Prompt removal of foreign bodies is crucial to prevent bowel wall injury. Improved clinical awareness, parental supervision, and diagnostic approaches are essential to enhance patient safety in cases of foreign body ingestion.

## Ethical approval

Ethical approval was not necessary for this case report as it involves the presentation of a single patient’s medical history and treatment. The case report does not involve experimental interventions or research on human subjects. It focuses on the clinical management and challenges associated with foreign body leading to complete small bowel obstruction. Therefore, ethical approval for this particular case report was not required.

## Consent

Written informed consent was obtained from the patient’s guardian as the patient is underage for filling the consent form. They gave consent for the publication of this case report and accompanying images. A copy of the written consent is available for review by the Editor-in-Chief of this journal on request.

## Sources of funding

No funding was received for the study.

## Author contribution

K.K.Y. had taken the history and was involved in the active management of the case. S.S., R.G., K.K., and R.K.Y. were involved in the writing of the manuscript. K.K.Y., J.K.S. and B.G. edited and revised the manuscript. All authors have read and approved the final version of the manuscript.

## Conflicts of interest disclosure

The authors have no conflicts of interest to declare.

## Research registration unique identifying number (UIN)

None.

## Guarantor

Krishna Kumar Yadav; Krishna321ydv@gmail.com.

## Data availability statement

All available data are within the manuscript itself.

## Provenance and peer review

Not commissioned, externally peer-reviewed.
